# Fulton County Medical Society

**Published:** 1871-02

**Authors:** Charles Pinckney


### THE GEORGIA MEDICAL COMPANION:

A MONTHLY ADVISER.

VOL. I. ATLANTA, GA., FEBRUARY, 1871. NO. 2.

### PART I.

### Original Communications and Special Selections.

### FULTON COUNTY MEDICAL SOCIETY, ATLANTA, GA.

*Extract from the Minutes of January* 20, 1871. *Reported by* CHARLES PINCKNEY, A. M , M. D.

Report of cases being in order, *Dr. W. T. Goldsmith* reported a case of Hysterical Mania, dependent upon engorgement and hypertrophy of the cervix uteri. Mrs. B., aged 57 years, and the mother of six children, had, since the birth of her last child—18 months—been the constant subject of various manifestations of hysteria. The history of the case, as given by herself and friends shows in a remarkable manner, the singular freaks of this peculiar malady. The patient attributed "all her woes" to the death of a brother, which occurred shortly after the birth of her last child. For some weeks subsequent she betrayed no apparent consciousness whatever of surrounding objects—failed to recognize the faces of her husband and most intimate friends, and in a semi-paralytic state, lay passive upon her bed, partaking of food barely sufficient to sustain life, and then only in deference to the most earnest entreaty. Emerging from this condition, she regained entirely the use of her muscular system, but with her intellectual faculties much weakened. At this time she was considerably reduced in flesh, had frequent "fits of crying," and occasional attacks of "hysterics." This state of things continued for several months, alternating between better and worse, until finally a periodic diarrhœa supervened, each attack of which would last for a week or more, and invariably give place to intense burning sensations in the lower extremities. This stage was sometimes accompanied with mania, as manifested by incongruity of conduct and wildness of speech. The patient was now, however, conscious of her discrepancies, and would remark that she had been acting and talking in a "foolish manner." Quite a number of medical advisers—regular and otherwise—had, from time to time, "worked upon" the case, without the slightest apparent success. It is said that "too many cooks spoil the broth." Perhaps the foregoing was an instance.

Such, then, is the history of this case up to the time I was called in. I immediately suspected that her symptoms depended, in a great measure, upon a diseased condition of the os uteri. Upon interrogating the patient and her friends, it was found that there had been no catamenial show since her last labor; that she had dull pain and heaviness in the pelvis; and, upon examining the abdomen, that there was a very painful spot over the right ovary. No tumor, however, could be felt. The spinal column exhibited signs of tenderness at various points. The finger, *per vaginam*, found the uterus prolapsed, and the os patulous and hypertrophied. The patient evinced inaccuracy of expression, strangeness of manner, and considerable deficiency of hearing. She complained of intensely burning sensations in her feet. Her friends declared that all these symptoms, together with insomnia, were at times wanting. At my next visit the speculum was introduced, and the cervix uteri found to be large, congested and inflamed, with several red spots surrounding the os. I prescribed the following treatment: hydrate of chloral, in fifteen grain doses, to be repeated every hour at night until sleep was induced; to be taken during the day whenever demanded by pain or excitement, and every night so long as insomnia should persist. Stimulating liniments to be applied to tender spot over right ovary. The cervix uteri was gently, but thoroughly touched with the solid nitrate of silver. These applications were repeated every 4

or 5 days. Hydrate of chloral having performed all that was expected of it, the following prescription I ordered given :

℞—Syr. Ferri Iodidi, . . . . ℥ i.

Tr. Acetæ Racemosæ, . . . ʒ v.

Tr. Rad Aconiti, . . . . ʒ ii.

Mix. S. 20 drops three times a day.

The patient desired some application to the feet, but was comforted with the assurance that the burning sensation would disappear under the treatment. The chloral acted charmingly in procuring sleep, in soothing pain, and quieting the excitability of the nervous system. Under the treatment instituted, improvement was immediate. The congested and inflamed os uteri yielded readily to the anti-phlogistic applications of nitrate of silver. The catamenia appeared, the diarrhœa ceased, the burning sensations vanished, and the patient, after months of suffering, is now in the enjoyment of excellent health.

*Dr. G. G. Crawford* reported a case of Strangulated Hernia, which was reduced by the administration of hydrate of chloral. After employing taxis and other means to no purpose, chloral ʒ ss. was given every two hours. The third dose produced sound sleep, relaxation and spontaneous reduction. He had seen the same thing occur after a large dose of opium.

*Dr. Goldsmith* said that he had once succeeded in reducing a strangulated hernia by the agency of cold water. In this case, opiates, taxis, warm water, nauseants, etc., had all failed to give relief. Having elevated the hips of the patient, a stream of cold water was poured upon the hernial tumor.

*Dr. W. G. Owen* said, he would state in this connection, that one evening during last year, he was called to see a little boy, who had been suffering for 24 hours with strangulated hernia. Having seen coffee highly spoken of in one of the journals, he concluded to try its effects in this case, and so directed. The beverage (made strong,) was given at intervals during the night. No other treatment was instituted. Spontaneous reduction occurred next morning.

*Dr. T. S. Powell* reported a case of shoulder presentation, which occurred about three months ago. The lady had been eight hours in labor with her second child, the arm of which was found presenting. The os uteri was about the size of a silver dollar. Dr. Withers was sent for and requested to administer chloroform. While under the anesthetic influence, turning was effected, and delivery by the feet accomplished in forty minutes. My object in presenting this case is to call attention to a single point, the measure of which cannot be overestimated. I mean the great importance of turning just as soon as the os is dilated sufficiently to admit the hand, thus anticipating impaction of the fœtal shoulder in the pelvic cavity. Hence version should be attempted at the very earliest practicable moment.

*Dr. E. J. Roach* reported a case of partial placenta previa, which occurred Nov. 1st, 1870. The force of the fœtal head was so great as to completely cut off the presenting portion of placenta, which, being otherwise detached, passed out like a clot. The part thus severed was about two inches wide, and four inches long. For upwards of four hours before the detachment of the interfering placental margin, there had been inertia of the womb, which finally succumbed to the free administration of brandy. But little hemorrhage occurred, though the woman was strong and plethoric. She had seven times before been a mother, and in a previous labor there was placenta previa, in which the child perished. In the case here reported, both mother and child did well.

*Dr. Goldsmith* said: I would like to enquire of members present their experience as to the per centage of fatality in cases of placenta previa?

*Dr. Powell :* It is laid down by all authors that no disease is more dangerous than complete placenta previa. It eclipses cholera. Simpson says: "there is no one complication in midwifery attended with more anxiety to the practitioner, and few, if any, with more real danger to the patient, than cases of unavoidable hemorrhage from presentation of the placenta." The celebrated Dr. Dewees observes that "no accident attendant on conception, is equally menacing as unavoidable hemorrhage, and it also emphatically declares to the physician that much depends on him, that it shall not be very often fatal. It is one of those extraordinary cases in which nature does less for the preservation of the individual than in almost any other." According to an extensive analysis of recorded cases, made by Dr. Simpson, out of 654

cases, 180 mothers were lost, being not quite one in three. It is more fatal than yellow fever or cholera, in their most malignant forms; and, according to the author last quoted, is "twice as dangerous and fatal as the operation of lithotomy." In my own experience, I have had four or five cases of placenta previa. In one instance the tampon was used, and ergot of rye given in large doses, as was done in a case reportod by Dr. Curry, of Knoxville, Tenn. When the pains became strong enough to force the fœtus powerfully upon the placenta, the latter was punctured, and the child came rapidly through the orifice. The presentation in this instance, was complete. I have never lost any cases. Simpson holds that it is proper to remove the placenta first—the hemorrhage being from that body itself, and in proportion to the amount of placenta detached.

*Dr. Pinckney:* The opinion of Drs. Churchill, Lee, Ashwell, and others, that hemorrhage proceeds from the uterine vessels, is, perhaps, correct. According to Churchill, "when the placenta reaches only the margin of the os uteri, it admits of the same treatment as accidental hemorrhage, for after rupturing the membranes, the pressure of the head while dilating the os uteri, will close the mouths of the bleeding vessels with the placenta, and so arrest the flooding until the child is expelled." What vessels? Evidently those of the womb. Dr. Lee says, "it escapes from the uterine sinuses laid bare by the detachment of the placenta." If such is not the case, I am at a loss to account for hemorrhage following the expulsion of the placenta entire. Whether there be direct vascular communication between the uterus and placenta is a question about which physiologists still differ. If there is not, I am unable to see how the loss of placental blood can so suddenly and alarmingly affect the mother, though I readily understand how it could quickly destroy the child. If there is, then it is not, I think, unreasonable to hold that, in placenta previa, the hemorrhage may proceed from the placental as well as uterine sinuses.

*Dr. Powell:* I was merely giving the opinion of another; not my own. I believe that hemorrhage proceeds from the uterus.

*Dr. Roach* stated, that out of three cases of placenta previa, occurring in his practice, one mother died and two recovered. In one instance, both mother and child were saved.

